# The Impact of COVID-19 Pandemic on Agricultural, Livestock, Poultry and Fish Sectors: COVID-19 Impact on Agriculture, Livestock, Poultry and Fish Sectors

**DOI:** 10.1155/2024/5540056

**Published:** 2024-10-29

**Authors:** Yashpal Singh Malik, Mohd Ikram Ansari, Rasha Gharieb, Souvik Ghosh, Ratan Kumar Chaudhary, Maged Gomaa Hemida, Dayan Torabian, Farzad Rahmani, Hadis Ahmadi, Pouneh Hajipour, Sina Salajegheh Tazerji

**Affiliations:** ^1^College of Animal Biotechnology, Guru Angad Dev Veterinary and Animal Sciences University, Ludhiana 141001, India; ^2^Department of Biosciences, Integral University, Lucknow, India; ^3^Department of Zoonoses, Faculty of Veterinary Medicine, Zagazig University, Zagazig 44511, Egypt; ^4^Department of Biomedical Sciences, Ross University School of Veterinary Medicine, P.O. Box 334, Basseterre, Saint Kitts and Nevis; ^5^Department of Veterinary Biomedical Sciences, College of Veterinary Medicine, Long Island University, Brookville 11548, New York, USA; ^6^Department of Clinical Sciences, Faculty of Veterinary Medicine, Science and Research Branch, Islamic Azad University, Tehran, Iran; ^7^Department of Clinical Sciences, Faculty of Veterinary Medicine, Karaj Branch, Islamic Azad University, Karaj, Iran; ^8^Department of Clinical Sciences, Faculty of Veterinary Medicine, Tehran University, Tehran, Iran

**Keywords:** agriculture, COVID-19, fish, livestock, pandemic, poultry

## Abstract

COVID-19 pandemic is considered a global crisis that adversely impacted the world economy. The virus possessed a serious threat to different sectors including agricultural, livestock, poultry and fish sectors in both developing and developed countries. COVID-19 pandemic and the associated lockdown for a long period have not only caused enormous distress to the millions of poor and marginal farmers for saving their crops and/or livestock but also affected livestock, poultry production systems and associated value chains, nutrition, health care and labour availability. In addition, COVID-19 pandemic significantly impacted the fishery sector through disruption in fish supply and value chains and had noteworthy effects on income of fish stakeholders, especially in developing countries. In this regard, the current review discussed the impact of COVID-19 pandemic on agricultural, livestock, poultry and fish sectors.

## 1. Introduction

The COVID-19 outbreak started in Wuhan, China, and became a global pandemic. With continuous increasing cases, the COVID-19 pandemic posed a significant threat to health with a ripple effect on various sectors impacting human life [1, 2]. To flatten the infection curve, the governments have imposed lockdowns, travels, trade restrictions and quarantines in most countries of the world's largest economies. Although the measures have a high level of efficiency in controlling the spread of COVID-19, they have also had an impact on several sectors: the agricultural, livestock, poultry and fish sectors [3]. This resulted in an economic crisis and recession due to the reduction in the workforce and loss of jobs across all sectors [4]. The agricultural sector has played a critical role in fostering the economy of different nations and is a source for early development in countries. With the ongoing COVID-19 pandemic, the agriculture sector faced huge challenges in satisfying the increasing demands for food. COVID-19 pandemic–induced lockdown had disturbed the agricultural sector, leading to a shortage of labour, an unavailability of agricultural inputs, an imbalance in crop production and farm gate prices, and issues with marketing and trading the agricultural output [5]. Consequently, the flow of food supply from farmers to consumers was disturbed, thus the world population faced food insecurity due to a reduction in food production, price hikes in the food market and reduced consumer purchasing power [6, 7].

Regarding the livestock production sector, restrictions of movement at national and international levels during COVID-19 pandemic have negative impact on livestock productivity and reproduction in livestock farms through reducing the availability of feed, medicine, farm inputs, veterinary services, farmworkers and increasing production and transportation costs [8]. The closure of processing plants, live animal markets, import and export restrictions delayed the marketing of livestock and livestock products, and this subsequently affected the income of livestock farmers, especially in low-income countries and threaten their livelihoods [9]. In addition, COVID-19 pandemic lockdown has disrupted the livestock supply chain and gravely reduced the consumer purchasing power, food demand and consumption [10].

Remarkably, the poultry sector in many countries worldwide has been negatively affected by COVID-19. In particular, the small-scale poultry production system in developing countries faced significant challenges during the disease outbreaks because most households in these countries raise poultry as a source of food (meat, egg) and income [11, 12]. COVID-19 and its related lockdown restrictions adopted by the governments disrupted the poultry supply chain including unavailability of farm inputs, an increase in production and operational costs, shortage of labour and human resources, reduced consumer demand and sales, and loss of farm finances [12]. Consequently, the livelihoods and food security status of poor and rural households in developing countries were affected.

On a global scale, the COVID-19 pandemic disrupted the fisheries at every step of the seafood supply chain, through lockdown measures, travel restrictions, trade stoppages and closure of hotels, restaurants and cafes. These impacts devastated the income and livelihood of fish farmers, fishers and all the people engaged in the aquaculture and fish sector in low- and middle-income countries [13]. In this context, the present review summarized the impact of COVID-19 on agriculture, livestock, poultry and fish sectors.

## 2. The Impact of COVID-19 Pandemic on Agricultural Sector

### 2.1. The Impact of COVID-19 Pandemic on Agricultural Production

The agricultural sector is associated with food production and represents the most integral domain of human growth [14, 15]. COVID-19 affected agricultural practices in two key areas: food supply and demand [16]. These two components are essential for food security, so food safety is at risk. The COVID-19–related lockdowns have direct or indirect consequences on crop production through reducing the availability of inputs (seeds and fertilizers), disrupting fertilizing, weeding, transport, processing services and controlling pests and diseases. This resulted in low agricultural production and affected planting as well as the quantity and quality of crop harvests [17]. In most countries in Africa, the COVID-19 pandemic–related lockdowns have not affected the crop production significantly but adversely affected the food security due to a negative impact on commodity supply chain, market availability, price volatility and farmers' incomes [17].

A substantial decline in the incomes of most developing countries due to COVID-19 outbreaks posed a serious threat to food security and livelihoods, where agricultural production systems are more labour-intensive and there is less capacity to withstand a severe macroeconomic shock [18]. Lockdowns and limits on the mobility of people across borders led to labour shortages in many countries characterized by labour-intensive production in agricultural sectors which resulted in low food production and food insecurity [19]. Thus, low-income households were more affected with COVID-19 because their income depends on agriculture sector [20].

The shortage of workers during the global COVID-19 pandemic affected the planting and harvesting of nonstaple crops like fruits and vegetables in Canada, the United States and Europe which have a shortage of nearly 1 million migrant workers from Eastern Europe and African countries [21]. The United Nations Conference on Trade and Development (NCTAD) report identified changes in the demands of all the commodities and reported a reduction in the price of agricultural commodities of 6.8%. Although the price of wheat increased by 4% in Canada, soybean and corn prices decreased by 8% and 10%, respectively [22]. A report in Southeast Asian countries estimated that agriculture production decreased by 3.11% (17.03 million tons) in the first quarter of 2020 due to the absence of labourers on the farm [23].

The Food and Agriculture Organization has detected several issues in the agricultural sector in different countries; the nationwide lockdowns associated with the COVID-19 pandemic forced the migrant workers in India to return to their homes and this disrupted the harvesting of winter crops due to a shortage of labour [24, 25].

**Figure 1:**
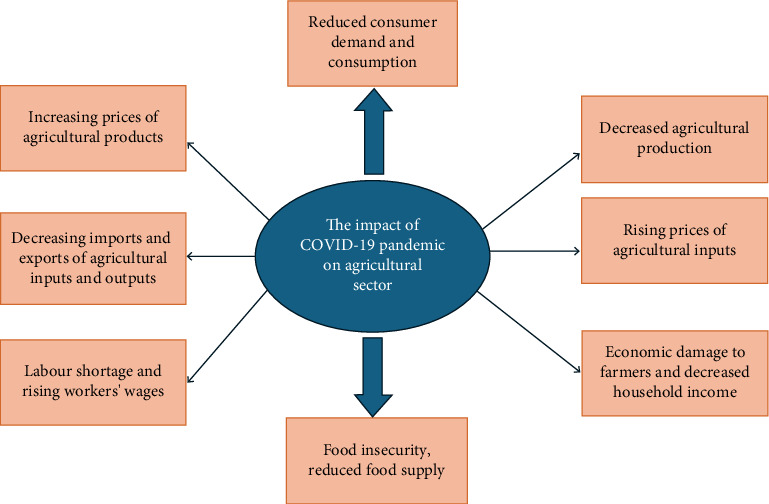
The impact of COVID-19 pandemic and lockdown on agricultural sector.

In Ethiopia, the farmer's income and production intentions were affected by the COVID-19 pandemic due to the overstocking of products and shortage of important inputs [26]. COVID-19–related lockdowns disrupted the export of rice from some countries such as India, Pakistan and Thailand, and this resulted in an increase in the price of rice in imported countries such as Iran by 13% and reduced its imports by 50% [27]. Other rice exporter countries such as Russia, Vietnam and Saudi Arabia limited rice and wheat export due to rising demand and declining seasonal harvest [28, 29]. Qualitative and quantitative analysis of the impacts of COVID-19 on the agricultural sector has been reported in different studies worldwide (Table 1, Figure 1).

### 2.2. The Impact of COVID-19 Pandemic on Food Security and Food Demand

Food security is defined as ensuring the availability and the accessibility of sufficient amount of nutrient-rich food to all communities [41]. The lockdown restrictions and economic crisis associated with COVID-19 posed a negative impact on food security particularly in developing countries by decreasing people's income, increasing food prices and reducing food purchasing ability. This resulted in (a) reducing access of food to the poorer segment of the population; (b) changing food consumption patterns; (c) excessive wastage of perishable products like fruits, vegetables and milks due to inefficient production systems and transportation restriction; (d) disruption of markets and improper public distribution system due to nonavailability of food stocks [42].

The COVID-19 pandemic had a significant impact on food demand, especially in developing and low-income countries. The reduction in consumer demand could be related to the declining in household incomes and the reduction of exports during COVID-19 lockdowns and restrictions [43].

## 3. The Impact of COVID-19 on Livestock Sector

### 3.1. The Impact of COVID-19 on Livestock Farming System

The livestock is a primary source of income and an essential source of food in many countries worldwide. Livestock production can be indirectly impacted by interruptions at processing and marketing levels. The measures undertaken by the governments during the COVID-19 pandemic including border lockdowns, movement restrictions and closure of markets resulted in potential overstocking and delayed marketing of finished animals [44].

The reduced access of livestock producers to farm inputs (animal feed and water), veterinary extensions and drug services during COVID-19 lockdowns posed a great threat to livestock productivity and resulted in disease outbreaks [45]. Globally, certain countries have banned export of livestock product, and this created a demand–supply gap in the animal feed industry. In addition, many of the feed industry in India depends on labourers, and the country-wide lockdowns caused shortage in labourers [46]. This puts the entire feed production industry in crisis and resulted in immediate shortage of animal feed supply. The limited access to the commercial pig feed due to transportation restrictions during the COVID-19 pandemic was a major issue for commercial pig production in India [47]. In China, COVID-19 transportation restrictions had a severe impact on livestock production sector as these restrictions led to shortage in the supply of livestock production materials (feed supplies) and rise in production cost [48].

Argentina and Brazil are a major beef producers and exporters, and the macroeconomic conditions caused by the COVID-19 pandemic affected the production and prices in these countries. Consequently, the national beef production in Brazil declined by 4.6% in 2020 and the local beef prices increased [49]. In Australia, the cattle herd declined 12% in 2020, and the sheep flock declined 9% from 70.6 million head in 2018 to 64 million head in 2020 [49].

COVID-19 restrictions have significant impacts on livestock vaccination and veterinary medicine. The rural community animal health workers were also unable to procure veterinary drugs and vaccines due to movement restrictions between towns. The vaccines and veterinary drugs were unavailable in some countries such as Jordan or India, Uganda, North of Cameroon and Central Africa [50]. The household livestock keepers in different countries worldwide reduced the number of owned animals due to increasing in prices of animal feed and drugs during the COVID-19 pandemic in 2020. In certain places in West Africa and India, the movement and border restrictions due to COVID-19 disrupted traditional migration patterns and restricted the access of livestock keepers and pastoralists to water and pasture as they depend on seasonal migration between grazing areas [51]. Subsequently, livestock household keepers in Africa lost 20%–40% of their income between March and May 2020 and farm-income earning opportunities for many pastoralists were inaccessible, thus hindering their ability to earn income from alternative livelihoods [52].

Algeria and Iran are largest sheep producers' countries, and the adverse economic and social consequences of the COVID-19 crisis affected the production and prices of sheep. The pasture-based production system in Iran accounts for nearly 60% of total sheep production, and this system exposed to droughts and diminished pastures especially in the last 2 decades. This resulted in increasing the production costs of sheep farming due to greater reliance on imported feed which become more expensive during the COVID-19 pandemic. These issues forced some sheep producers out of business, and consequently, the national sheep flock reached its lowest level in 2020, 12% below 2014 levels [53]. In India, the COVID-19–induced lockdown caused economic losses of 32.4 million United States (US) dollars to the livestock production sector [54].

In Morocco and Jordan, the livestock production was influenced by the COVID-19 pandemic led to a decline in domestic sheep meat consumption in both countries. In Morocco, low domestic demand for sheep meat in 2020 prevented producer prices from keeping up with increased feed prices. However, the domestic demand for sheep meat was low in Jordan and the sheep meat producer prices increased by 10% in 2020 [55].

### 3.2. The Impact of COVID-19 on Milk Production

**Figure 2:**
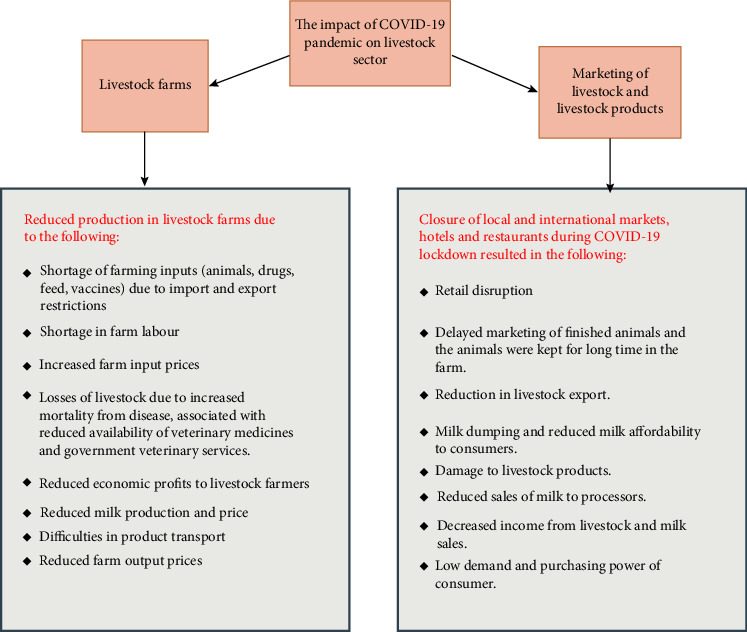
The impact of COVID-19 pandemic and lockdown on livestock sector.

The milk production decreased in some countries during COVID-19 lockdowns due to some factors; unavailability of dairy meal and other concentrates forced the farmers to use local and low-quality homemade rations, the incidence of diseases and the lack of veterinary services (drugs and vaccines) reduced cow productivity. The milk collection from farmers decreased because many farm household members including children were forced to stay at home, resulting in less milk available for sale. For instance, COVID-19 lockdowns affected about 1.5 million Indian dairy farmers who reported a decrease in the milk volume from 545 thousand litres per day before the lockdown to 200–375 thousand litres a day during the lockdown [56]. The dairy sector in China was greatly affected by COVID-19 lockdowns due to production and transportation issues. An insufficient supply of farm inputs, an increase in their prices and lack of labour adversely affected the milk production in China [57]. The impact of COVID-19 on dairy production in different countries is listed in Table 2 and Figure 2.

### 3.3. The Impact of COVID-19 on Marketing of Livestock and Livestock Products

COVID-19–related market closures in many countries have reduced the sales of live animals and the opportunities for livestock products to enter the market for consumers to purchase. The disruption in marketing processes in different countries during the COVID-19 pandemic in 2020 resulted in the delayed marketing of finished animals, monthly volatility in finished animals' prices and declines in producer prices (farm gate prices) in 70% of countries worldwide [64].

In low-income countries, livestock farming occurs in traditional small-scale systems, and the reduced market opportunities threaten farmers' livelihoods [65, 66]. Thus, the livestock keepers and pastoralists were unable to sell animals in live animal markets and this caused the loss of crucial annual income and inability to buy food, other basic needs and animal healthcare services, leading to increased livestock mortality [67]. In China, livestock enterprises and farmers failed to deliver livestock products to the market during COVID-19 lockdowns that lead to the interruption of the entire livestock industry supply chain [68].

In the United States, the total consumption of beef products increased by 0.89% during the COVID-19 pandemic in 2020 and this was not sufficient to absorb huge supplies of cattle and beef in the market due to shrinkage of exports (4.9%) and weak demand in key export markets (Japan, South Korea and Mexico). Consequently, beef producer prices in the United States declined by 7% in 2020 [69, 70].

According to the Famine Early Warning Systems Network [71], 55%–70% of Somali annual livestock are exported to Saudi Arabia to satisfy the demand during the Ramadan and Hajj seasons. However, the COVID-19 pandemic caused sharp decline in livestock exports from Somali to Saudi Arabia in 2020 and 2021. The communities involved in Hajj export trade including pastoralists, brokers, vehicle hire operators, small- and medium-sized enterprises and exporters were also adversely affected by COVID-19–related border lockdowns [72].

In several rural areas in India, the milk prices reduced by 50% due to the unavailability of milk cooperatives or any private agencies for marketing [73, 74]. In Canada and the United States, the COVID-19–related closure of retail shops, markets and lack of processing facilities forced the dairy farmers to dump fresh milk due to the inability to store milk for many days resulting in economic losses [75, 76]. In Bangladesh, dairy producers could not sell the milk in marketplaces due to COVID-19 lockdowns. Subsequently, the fresh milk was discarded, and the prices have drastically declined resulting in economic losses of about 67 million US dollars [77, 78]. In Pakistan, dairy farmers could not sell approximately 57.3 billion litres of milk and other dairy products due to COVID-19–related markets' closure [79]. The impact of COVID-19 on the marketing of livestock and livestock products in different countries is listed in Table 2 and Figure 2.

## 4. The Impact of COVID-19 Pandemic on Poultry Sector

**Figure 3:**
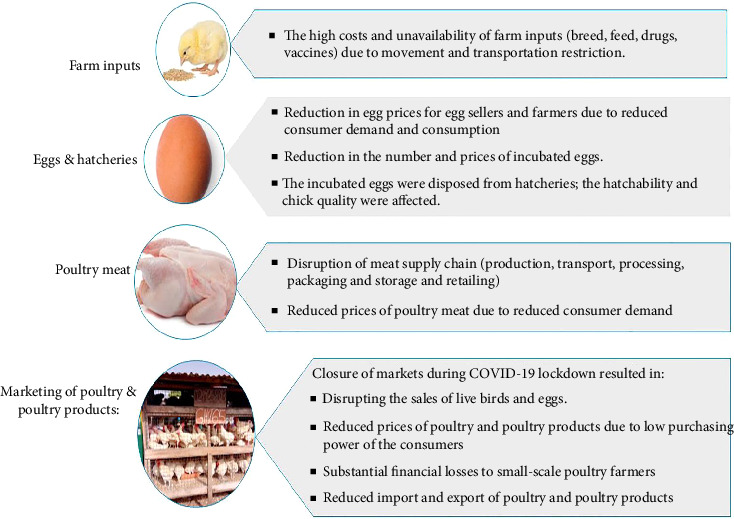
The impact of COVID-19 pandemic and lockdown on poultry sector.

Poultry diseases are the main issue for the future strategy of the poultry industry. Although poultry are not reservoirs for SARS-CoV-2, the COVID-19 disease has adversely affected the poultry industry worldwide because of the lockdown and limitations imposed to control the spread of the virus [80].  Figure 3 shows that the COVID-19 pandemic and the related lockdowns brought an unprecedented impact on poultry sector due to the high cost and unavailability of feed ingredients, shortage of labour, inadequacy of vaccines in the local market, lack of veterinary services, low purchasing capacity of the consumers, continuous price fluctuations in the retail and wholesale market [8]. The high costs and unavailability of poultry feed could be related to that some countries such as Argentina and Brazil prevented the export of raw material of feed: soybean and corn, which led to a shortage of poultry feed in most countries [81, 82].

India is the fourth-largest poultry producers in terms of volume. The post-COVID-19 lockdown impacted the production in the broiler and layer farms in India with losses estimated as 3.23 billion US dollars. The prices of live poultry birds were low as 0.12–0.36 US dollars/kg, especially in the first week of lockdown when the poultry farmers could not connect to the markets [83]. A study conducted by Mishra et al. [84] reported that backyard poultry farmers in Gujarat, India, did not suffer any financial loss or problems in procuring poultry feed during the COVID-19 lockdown since the farmers did not buy any feed from outside and the birds were fed on what available at home. The authors also reported that the farmers did not face any problems with the supply of medicine to backyard birds.

In Bangladesh, Fouzder et al. [85] studied the impact of COVID-19 on smallholder poultry farms in the southern part of Bangladesh, the authors reported great losses in broiler production during the COVID-19 pandemic, and most farmers stopped their business because of rises in prices of farm inputs (feed, chick and medicine), decline in the consumers' demand for poultry and poultry products and low prices of live birds in the market. Furthermore, Amin et al. [86] studied the influence of COVID-19 on poultry and poultry products in Bangladesh. The authors declared that many smallholder poultry farmers and hatchery owners were forced to shut down in the early phase of COVID-19 due to less/no demand of poultry and their products along with high cost of poultry feed, unavailability and high cost of poultry medications and vaccines in local markets, lack and high cost of transportation, lack of extension services, fluctuation in the demand and market prices corruption in purchasing raw materials and distribution of poultry products and loss of farm income.

In Italy, COVID-19 lockdown had a significant negative effect on poultry sector. The country outlawed the import of poultry meat which has resulted in a 15%–25% decline in chicken consumption. In addition, chicken hatcheries were severely affected, and several hatcheries have been obliged to put birds to sleep before reducing the quantity of eggs being incubated [87]. The eggs in hatcheries were kept for a longer period at low temperatures, and this resulted in a decreasing number of incubated eggs. Thus, the hatchability and chick quality were diminished over time.

In Egypt, broiler production systems are classified into three main categories: industrial production systems, small-scale (smallholders) commercial production systems and backyard production systems. Statistics of Egyptian Ministry of Agriculture and Land Reclamation [88] showed that small-scale commercial systems represent around 70% of all poultry-producing farms in Egypt. Poultry production systems in Egypt depend mainly on imported feed raw materials, feed additives, vaccines, veterinary supplies and production equipment. The availability and prices of these inputs were greatly affected by the COVID-19 pandemic. Abu Hatab et al. [89] studied the impact of COVID-19 on 205 small-scale commercial broiler farms consisting of both farm-based and household-based production systems in Egypt. The results reported that COVID-19 affected all production systems through: (1) unavailability and high costs of production inputs (chicks, feed, vaccines, veterinary medicines and equipment), (2) increased mortality rates in birds, (3) increased cost of transportation, (4) lay off workers and loss of skilled labour, (5) decreased worker productivity by the reduction in the number of working hours and working days, (6) increased cost of wages, (7) difficulties in access to markets and supplying products to local markets, (8) reduced the market demand (retailers and consumers) and sales, and (9) reduced farm financial services. Remarkably, the authors stated that farm-based systems were 2.38 and 2.34 times more likely to perceive COVID-19 impacts on the availability of production inputs, labour and human resources than household-based systems. On the contrary, household-based systems were less likely than farm-based systems to perceive the impact related to farm production and operational costs. This could be attributed to the characteristic small size of household-based farms, their reliance on family labour and selling directly to consumers without incurring transport costs.

In Indonesia, the poultry sector particularly smallholder chicken production system supports the lives of millions of Indonesians by offering employment opportunities and additional income to numerous smallholders in rural regions [90]. The outbreak of the COVID-19 pandemic in early 2020 exposed the poultry sector in Indonesia to unexpected challenges, and the impact was not severe in smallholder farms as they depend on family labour [91]. However, these farms faced unpredictability of the market price, uncontrolled variable cost of several essential farm inputs, such as feed, financial constraints because they could not adopt alternatives like distribution channels, online sales platforms and biosecurity to protect their flocks compared to larger farms. Kusumaningrum et al. [92] studied the impact of COVID-19 on smallholders' layer chicken farms in three different provinces in Indonesia, and the authors reported a significant increase in operational costs, specifically feed and labour as well as significant decrease in profit level during the COVID-19 pandemic. Additionally, Nendissa et al. [93] and Sembada, Daryanto and Andik [94] reported a dramatic drop in the supply chain, market price and consumer demand for broiler chicken in Indonesia during COVID-19 and the economic growth fell from 4.97% to 2.97%.

In Nigeria, the COVID-19 lockdowns caused a devastating effect on large-scale poultry production quantitatively and qualitatively through unavailability of product inputs, increasing the production cost and disrupting transportation and sales [95]. Bamidele and Amole [96] studied the impact of COVID-19 on smallholder poultry farms in 5 states of Nigeria. The authors reported that most households relied more on chickens for food and income, and they suffered from a significant reduction in monthly income during COVID-19 pandemic. Furthermore, most households reported an increase in flock size during the pandemic through the hatching of chicks by local hens. In addition, COVID-19 restrictions and lockdowns reduced the farmers' access to markets for the sale of live birds and eggs.

In Myanmar, the COVID-19 lockdowns resulted in closure of approximately 30% of broiler farms and 10% of layer farms; reduction in chicken meat and egg prices; reduction in the demand for chicken meat by 60% for broiler and 40% for layer; and layoff of 42% of long-term farm workers [97].

## 5. The Impact of COVID-19 Pandemic on the Fisheries and Aquaculture Sector

In many countries, fish production is considered an essential activity contributing to income, household resilience, trade and food security. The lockdowns implemented by some countries during COVID-19 pandemic have resulted in logistical difficulties in the seafood trade, particularly in relation to transportation and border restrictions [98]. Fishery production in some countries has been significantly reduced during COVID-19 due to shortages of farm input supplies (seeds, feed and vaccines) which resulted in higher costs of inputs supplies as well as greater risk of fish mortalities [99]. Furthermore, fresh fish and shellfish supply chains and consumer demand were severely impacted during COVID-19 pandemic due to the closure of global markets and the food service sectors (e.g., hotels, restaurants and catering facilities, including school and work canteens) [100]. This resulted in an increase in live fish stocks and unsold fish products due to the reduction in fresh fish sales from established retailers. In some countries, particularly in less developed countries, wholesale and retail fish markets have become highly regulated to secure physical distancing and other sanitary rules, which indirectly limited the access of consumers to the market and thus reduced the income for fish traders and fishers [101]. Qualitative and quantitative analysis of the impacts of COVID-19 on the fish sector has been reported in different studies worldwide (Table 3).

## 6. The Established Strategies and Measures to Cope With COVID-19 Pandemics

• The governments supported the food and agricultural systems during pandemics through strengthening domestic markets and promoting intraregional trade.
• The governments and development partners facilitated market access for the producers and monitored the cross-border trade and the export of livestock, poultry, fish and their products.
• The e-commerce platforms and the use of digital tools were promoted to reduce physical contact and help the marketing of products.
• The governments in many countries regulated the supply and value chains in all sectors by developing road, market infrastructure, cold storage facilities, transport systems and farmer-to-market linkages.
• National/State Disaster Management Authorities were created to monitor animal health and ensure animal welfare measures during pandemics.
• Public distribution of systems for supply of animal feed/fodder and medicines in remote areas.
• Arrangement for vaccination of livestock animals and poultry against enzootic diseases during pandemic threats.
• The agencies monitored the transboundary animal movements during pandemics.
• All states' veterinary departments across the countries conducted skill development programmes linked to One Health Mission to train the veterinarians to assist in animal and animal products supply chain.
• The vet care helpline services were opened at district and statewide to pass on important animal advisories.
• The veterinarians and other vet service providers were vaccinated against COVID-19 during the pandemics.
• Adequate hygiene was ensured to the workers in all sectors following the WHO COVID-19 prevention guidelines through social distancing at workplaces, provision of handwashing points, wearing facemasks during handling animals, poultry, fish and their products.

## 7. Conclusion

COVID-19 pandemic disturbed the agricultural, livestock and poultry sectors. Despite the steps taken by the governments were essential for protecting the public health, the lockdown particularly caused economic collapse in all sectors as it disrupted the production and supply chain, limited import, export operations and marketing capacity, affected the global food security and changed the consumers' demand and behaviour. In this context, the governments should adopt innovative policies and strategies to protect the global food supply chain and achieve economic recovery during COVID-19 pandemic. For instance, the government should create congenial environment for reviving all affected sectors, ensuring the livelihood of the associated stakeholders and providing food to vulnerable populations during the pandemic. In addition, trade restrictions should be removed for food trade to improve international collaboration for food security.

## Figures and Tables

**Table 1:** The impact of COVID-19 pandemic on agricultural sector (case studies and reports).

Country	Affected parameters	Reference
Myanmar, Southeast Asia	• Delayed land operation	[30]
	• Reduced availability of machines	
	• Disruption in input retailers	
	• High prices of fertilizers and pesticides	
	• Hindered crop traders, difficulty in selling crops due to lower crop prices, poor demand, no buyers	
	• Reduced market access, market closure, no means of transportation to markets	
	• Decreased export	
	• Movement restrictions of farmers	
	• Reduced farm household income	

Burkina Faso	• Decreased availability and supply of agriculture input (seed and fertilizer)	[31]
	• Disruption in the availability of agricultural workers and off-farm income-generating activities	
	• Low producer prices for cottonseed and disruptions in transportation (from villages to ginning plants)	
	• Socioeconomic impacts, including lower farm and nonfarm incomes	

Central and Southern Africa	• The regional agricultural production season was not significantly affected by COVID-19	[32]
	• Planting for the 2020 main season started well before the COVID-19 outbreak	
	• The harvest was ongoing by the time movement restrictions that were adopted	

Somalia	• Lack of income to hire labour and buy seeds and fertilizer	[33]
	• Marketing difficulties due to low prices and demand and high transportation costs	
	• The overall incomes had decreased compared to the same time the previous year	
	• Farm households involved in nonfarm activities were more likely to report income declines than households involved in cropping, livestock or agricultural labour	

Sudan	• Movement and other restrictions affected the agricultural value chain (from producers to consumers)	[34]
	• Farm and agropastoral communities are among the most affected population groups	

Ethiopia, Kenya, Malawi and the United Republic of Tanzania	• A decrease in their participation in farming and other business activities	[35]
	• Severe disruptions to local labour markets	

Kenya	• Difficult access to inputs	[36]
	• The workers' movement across the borders was facilitated with special permits	

South Africa	• Labour shortages in the farming sector during the harvesting	[37]
	• Period, these challenges did not significantly reduce the expected maize output	

Northern Thailand	• COVID-19 negatively affected the daily lives of the farmers	[38]
	• COVID-19 increased the cost of planting and the cost of agrochemicals and fertilizers	
	• It also decreased the prices of agricultural products and agricultural extensions	
	• The markets and logistics of agricultural products during the pandemic were more difficult than before	
	• Half of the farmers had moderate stress	
	• The loss of household income increased household expenses	

India	• Change in overall prices of agricultural commodities	[39]
	• Decrease in the availability of agricultural inputs	
	• Difficulties in marketing and selling of agricultural production	

Karamoja, Uganda	• Marked decrease in land cultivated	[40]
	• Rise in the cost of agricultural inputs	

**Table 2:** The impact of COVID-19 pandemic on livestock sector (case studies and reports).

Country	COVID-19 impact	Reference
Kenya	• Increased cost of production and decreased productivity per cow	[58]
	• Disruptions in transportation, trade and access to dairy inputs	
	• Loss of income and employment and reduced milk affordability to consumers	
	• Low governance and decreased participation and coordination of stakeholder platforms	


USA, Canada, Australia, Brazil, South Africa, China, Argentina and others	• High monthly volatility of finished animal prices	[59]
	• Delayed marketing of finished animals	
	• Reduced producer prices	
	• Increased farm input prices	
	• Shortage in farm labour	
	• Reduced access to farm inputs	
	• Reduced feed supply	
	• Reduced access to service (veterinary extensions)	
	• Reduced pastoralists mobility	
	• Reduced national livestock inventories	

China	• Reduced the revenue of the livestock industry	[60]
	• Reduced livestock product sales	

Uganda	• Failure to sell animals	[61]
	• Increasing transportation costs	
	• Reduced number of cattle buyers	

Karamoja, Uganda	• Decrease in livestock prices	[40]
	• Losses of livestock due to a resurgence of raids	
	• Reduced milk availability	
	• Losses of livestock due to increased mortality from disease, associated with reduced availability of veterinary medicines and government veterinary services	
	• Difficult access to veterinary medicines and services	
	• Increased prices of veterinary medicines and services combined with low purchasing power	

West Africa	• Reduced livestock mobility and abnormal concentrations of livestock	[62]
	• Livestock market impact and reduction in livestock export	
	• Shortage of livestock feed	

Bangladesh	• Reduced milk production	[63]
	• Decrease income from milk sales	

China	• Insufficient supply of farm inputs	[57]
	• Increased price of farm inputs	
	• Lack of sufficient labour	
	• Difficulty in product transport	
	• Reduced milk production	
	• Reduced milk price	
	• Milk dumping	
	• Reduced sales of milk to processor	

**Table 3:** The impact of COVID-19 pandemic on fish sector (case studies and reports).

Country	COVID-19 impact	Reference
Sri Lanka	• Reduction of fish production	[102]
	• Unavailability of labour force	
	• Decline in stakeholder income	
	• Reduction in fish prices and sales	
	• Dramatic changes in consumers' consumption of fish and fish products	

Katabon, Dhaka in Bangladesh	• Decline in daily sales of aquarium fish stakeholders, including wholesalers, retailers, importers and hatchery owners	[103]
	• An increase in the prices of ornamental fish species ranged from 13.93% to 91.11%	
	• Reduced number of fish species in all aquarium stores	
	• Change in fish demand, the demand increased for goldfish and decreased increased for other species	
	• Aquarium fish imports declined from 30% to 2%, while local sources, particularly hatchery owners, increased from 70% to 98%	
	• The demand for aquarium fish species declined from 90% to 60%	
	• Reduction in purchasing power of fish feed (80% − 30%), medicine (60% − 20%) and other aquarium supplies (50% − 20%)	
	• An increase in transportation costs from 6.25% to 33.3%	
	• Significant losses in suppliers to the suppliers of the ornamental fish industry	
	• Significant changes in the aquarium fish marketing channel	

Mymensingh, Rajshahi, Rangpur, Khulna and Barisal districts in Bangladesh	• Lack of access to farm inputs (feed, fingerling, labour, ice, fishing gear, fertilizer, medicine) and change in their prices	[104]
	• Labour shortage	
	• High transportation costs	
	• Changes in fish prices	
	• Disruption in fish supply chain	
	• Disruption of value chain activities	
	• Limited number of fish buyers and market disruption	
	• Reduced consumer demand	
	• Decline in fish farmers' and traders' income from fisheries by 50%, while fisher's income dropped by 32.80%	

Brahmanbaria subdistrict, Bangladesh	• Unavailability of farm inputs	[105]
	• Labour shortage	
	• Disruption in transportation of fish fry and fingerlings and higher transportation costs	
	• Decline in the demand and prices for fish fry and fingerlings	
	• Breakdown in fish hatchery operations	
	• Inability to sell fish fry due to closure of public transportation	
	• Decline in the income of fish sellers	

## Data Availability

The data used to support the findings of this study are included within the article.
